# Insulin signalling and GLUT4 trafficking in insulin resistance

**DOI:** 10.1042/BST20221066

**Published:** 2023-05-30

**Authors:** Julian van Gerwen, Amber S. Shun-Shion, Daniel J. Fazakerley

**Affiliations:** 1 Charles Perkins Centre, School of Life and Environmental Sciences, University of Sydney, Sydney, NSW 2006, Australia; 2 Metabolic Research Laboratories, Wellcome-Medical Research Council Institute of Metabolic Science, University of Cambridge, Cambridge CB2 0QQ, U.K.

**Keywords:** glucose transport, GLUT4, insulin resistance, insulin signalling, phosphoproteomics, trafficking

## Abstract

Insulin-stimulated glucose uptake into muscle and adipose tissue is vital for maintaining whole-body glucose homeostasis. Insulin promotes glucose uptake into these tissues by triggering a protein phosphorylation signalling cascade, which converges on multiple trafficking processes to deliver the glucose transporter GLUT4 to the cell surface. Impaired insulin-stimulated GLUT4 translocation in these tissues underlies insulin resistance, which is a major risk factor for type 2 diabetes and other metabolic diseases. Despite this, the precise changes in insulin signalling and GLUT4 trafficking underpinning insulin resistance remain unclear. In this review, we highlight insights from recent unbiased phosphoproteomics studies, which have enabled a comprehensive examination of insulin signalling and have transformed our perspective on how signalling changes may contribute to insulin resistance. We also discuss how GLUT4 trafficking is disrupted in insulin resistance, and underline sites where signalling changes could lead to these trafficking defects. Lastly, we address several major challenges currently faced by researchers in the field. As signalling and trafficking alterations can be examined at increasingly high resolution, integrative approaches examining the two in combination will provide immense opportunities for elucidating how they conspire to cause insulin resistance.

## Introduction

**Figure 1.**
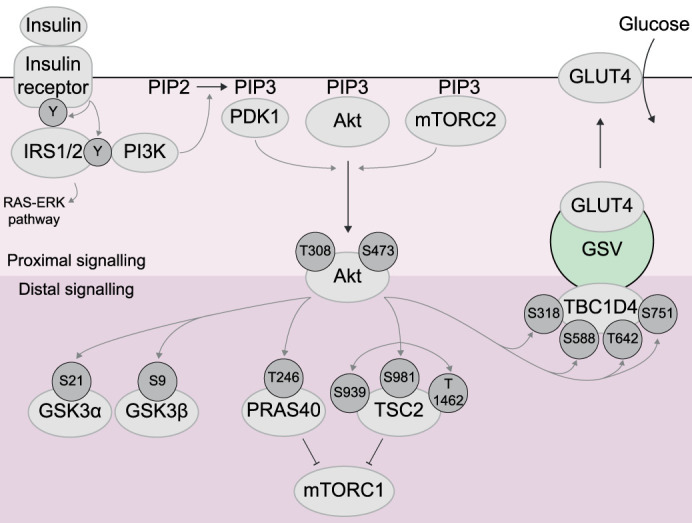
Overview of insulin signalling. Insulin initiates a protein phosphorylation cascade to regulate cellular metabolism (reviewed in [125] and [126]), with the kinase Akt as a central node in this cascade. The events leading to Akt activation — ‘proximal insulin signalling' — begin with insulin binding to its receptor, activating its receptor tyrosine kinase activity and triggering its auto-phosphorylation. Phosphorylated tyrosine residues bind the insulin receptor substrate proteins (IRS1 and IRS2), which are also phosphorylated, recruiting the lipid kinase PI3K to generate PIP3 at the plasma membrane. PIP3 then recruits additional kinases, including Akt, PDK1, and mTORC2, through PIP3-binding domains. PDK1 and mTORC2 sequentially phosphorylate Akt at T308 and S473, leading to full activation. In ‘distal insulin signalling', Akt phosphorylates multiple proteins to enact complex cellular changes. A primary function of insulin in skeletal muscle and adipose tissue is promoting the translocation of GLUT4-storage vesicles (GSVs) to the plasma membrane (adipose) and sarcolemma and transerve tubule membrane (muscle), with the best characterised regulator being the Akt substrate TBC1D4. Other Akt substrates include the kinases GSK3α and GSK3β, which promote glycogen synthesis, and PRAS40 and TSC2, which promote protein synthesis by relinquishing their inhibition of the kinase mTORC1. Insulin also activates Akt-independent signalling axes such as the RAS-ERK pathway [22], though Akt signalling is generally considered the major mediator of insulin's acute metabolic actions.

The peptide hormone insulin plays a key role in maintaining glycaemia. Insulin enhances glucose uptake into skeletal muscle and adipose tissues by activating the PI3K/Akt signalling pathway, regulating GLUT4 trafficking from intracellular compartments (known as the GLUT4-storage compartment (GSC), or GLUT4-storage vesicles (GSVs)) to the plasma membrane (PM) in adipocytes, or sarcolemma and transverse tubule membrane in myocytes (Figure 1).

The importance of GLUT4 in muscle and adipose tissues for whole-body glucose homeostasis is evident in knockout mouse studies [1,2]. Furthermore, impaired insulin-stimulated glucose transport into muscle is an early event in the progression to type 2 diabetes in humans [3]. Impaired insulin-stimulated glucose transport (insulin resistance) is thought to be due to reduced GLUT4 delivery to the cell surface, which has been measured in both human muscle and adipose tissue [4,5]. It is important to understand precisely how the insulin-GLUT4 pathway is perturbed in insulin resistance since insulin resistance is a major risk factor for the development of type 2 diabetes and other metabolic diseases, and there are currently no pharmaceutical interventions that target this pathway directly.

This review explores the molecular basis of insulin resistance in muscle and adipose tissue. Other recent reviews have focused on early events in the path to insulin resistance in these tissues, including organismal changes such as obesity and hyperinsulinemia [6,7], as well as cell stressors such as ectopic lipids and reactive oxygen species [6,8]. Here, we focus further downstream, on the insulin signal transduction machinery and the GLUT4 trafficking processes that it regulates. We will examine: (1) alterations to insulin signalling during insulin resistance; (2) changes to GLUT4 traffic in insulin resistance; (3) connections between signalling alterations and disrupted traffic; and (4) potential directions for future studies exploring the impaired insulin-GLUT4 pathway. This review is particularly timely given recent global phosphoproteomic analyses providing new insights into dysregulation of the insulin signalling pathway.

## Insulin signalling

### Is insulin resistance caused by a proximal signalling defect?

A prevailing hypothesis suggests that insulin resistance results from impaired proximal insulin signalling, leading to reduced Akt activation [6–8]. Indeed, rare deactivating mutations in Akt or upstream proteins cause insulin resistance [9] and reduced activation of Akt or its upstream proteins has been observed in insulin-resistant muscle and adipose tissues of mice [10,11], rats [12] and humans [13–19]. A proposed mechanistic basis for this hypothesis is that IRS1/2 and the insulin receptor harbour inhibitory phospho-serine/threonine residues targeted by multiple kinases. These kinases include JNK, p38, p70S6K, mTORC1, and canonical and novel PKCs, which are activated by molecular insults that trigger insulin resistance [20–22].

While these molecular events can occur, evidence suggests that defects in proximal insulin signalling are not the major determinant of insulin resistance. First, insulin resistance has been observed without decreased phosphorylation of Akt at its activating phosphosites in diverse models of insulin-resistant 3T3-L1 adipocytes and L6 myotubes, and human muscle *ex vivo* and *in vivo* [23–29]. Furthermore, in progressive high-fat feeding of mice, phosphorylation was decreased only after the onset of overt muscle insulin resistance [24]. Second, only a small subset of the total cellular pool of Akt needs to be active to achieve maximal substrate phosphorylation, as demonstrated in 3T3-L1 adipocytes and mouse muscle [24,30]. Therefore, decreased Akt phosphorylation in insulin resistance may not result in reduced substrate phosphorylation, as observed in human muscle [16]. Finally, the requirement for insulin receptor/IRS in insulin resistance has been tested through independent PI3K/Akt pathway activation via PDGF receptor overexpression. Here, PDGF-driven glucose uptake in isolated muscle or GLUT4 translocation in myotubes and adipocytes cells was impaired similarly to insulin-driven responses, except in myotubes made insulin resistant through chronic insulin exposure [24]. Given these findings, which have been reviewed in greater detail elsewhere [6–8], it seems unlikely that proximal insulin signalling is the key determinant of insulin resistance. This underscores the urgent need to examine a broader spectrum of insulin signalling nodes.

### Phosphoproteomics studies of signalling alterations in insulin resistance

Advances in mass spectrometry-based phosphoproteomics now enable the unbiased exploration of signalling in health and disease [31]. In particular, recent phosphoproteomics studies have examined insulin signalling changes in insulin-resistant myoblasts derived from stem cells of human subjects (iMyos) [32,33], in insulin-resistant murine cultured adipocytes and adipose tissue [34], and across skeletal muscle biopsies of adults with differing insulin sensitivity [35]. One of the most striking observations from these studies is that only a small fraction of the signalling changes seen in insulin resistance involved canonical insulin signalling proteins [32–34]. Moreover, these changes were inconsistent with a simple defect in proximal insulin signalling, as only a subset of Akt substrates showed impaired insulin responses [32–34]. Thus, global phosphoproteomics studies suggest that understudied branches of insulin signalling may be major mediators of insulin resistance.

**Figure 2.**
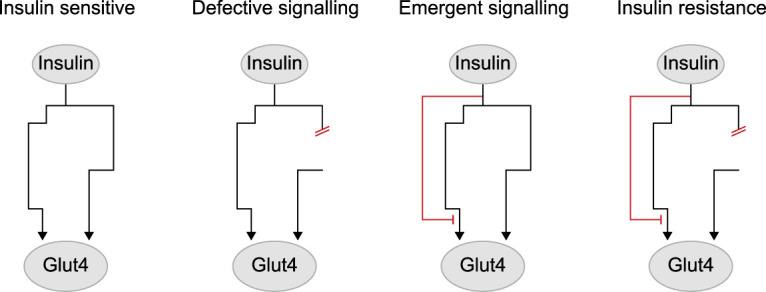
Defective and emergent signalling in insulin resistance. Phosphoproteomics has revealed that insulin resistance can be accompanied by both impaired insulin signalling responses (‘defective signalling') and the emergence of enhanced or novel insulin signalling responses (‘emergent signalling'). Both of these processes could contribute to insufficient insulin-stimulated GLUT4 translocation in insulin resistance.

These studies have also provided systems-level insights achievable only with unbiased omics technology. For example, in adipocyte insulin resistance, insulin-regulated protein dephosphorylation was preferentially impaired compared with phosphorylation [34], suggesting that there is dysregulation of insulin-activated protein phosphatases and/or insulin-deactivated kinases in insulin resistance. Additionally, while insulin resistance is classically thought to result from defective insulin signalling, phosphoproteomics has uncovered numerous emergent phosphosites, featuring enhanced or novel insulin responses in insulin resistance (Figure 2) [32–34]. This is exciting as previous research found that insulin resistance may involve the emergence of an insulin-activated negative feedforward loop targeting signalling downstream of Akt. In particular, Ng et al. demonstrated that chronic insulin and dexamethasone-induced insulin resistance in 3T3-L1 adipocytes can be overcome by activating Akt independently of insulin through drug-inducible heterodimerisation with a membrane-localised protein, but is reinstated by co-administration of insulin [36]. Overall, insulin resistance involves complex signalling rearrangements, with both defective and emergent signalling events potentially mediating impaired GLUT4 translocation (Figure 2).

Mechanistic validation of phosphoproteomic signatures has begun to pinpoint the specific signalling nodes driving insulin resistance. For example, deactivation of the kinase GSK3 by insulin was impaired in multiple 3T3-L1 adipocyte and mouse adipose tissue models of insulin resistance, and was not entirely attributable to reduced deactivation by Akt [34]. Acute GSK3 inhibition partially restored insulin sensitivity in these models, implicating GSK3 dysregulation as a mediator of adipocyte insulin resistance. Pharmacological inhibition and siRNA-mediated knockdown also revealed that the kinases MARK2 and MARK3 antagonise GLUT4 translocation in 3T3-L1 adipocytes [34]. Furthermore, activation of the RhoA GTPase, which regulates glucose uptake through cytoskeletal rearrangements, differed in iMyos cells in an insulin resistance and sex-dependent manner, in line with altered phosphorylation of RhoA regulators [33]. Interestingly, another Rho GTPase, Rac1, promotes GLUT4 translocation by regulating actin dynamics through a signalling axis parallel to Akt [37], and phosphorylation of the Rac1 target PAK was impaired in insulin-resistant muscle from mice and humans [38]. Finally, phosphorylation of S377 on AMPKα2 associated with glucose uptake in insulin-stimulated and/or exercised skeletal muscle from individuals with differing muscle insulin sensitivity, and was necessary for proliferation of MEF cells during glucose deprivation, suggesting a regulatory role in glucose metabolism [35]. However, a mechanistic link to insulin sensitivity is yet to be established for this site.

From the vantage point provided by phosphoproteomics, it appears likely that multiple, potentially independent signalling alterations mediate insulin resistance. These alterations could involve both defective and emergent signalling, originate from kinases including GSK3 and AMPK, and converge on functional effectors such as Rho GTPases. Comprehensively charting the signalling alterations that impair GLUT4 translocation and unravelling how they do so remains a significant task.

## GLUT4 trafficking in insulin resistance

**Figure 3.**
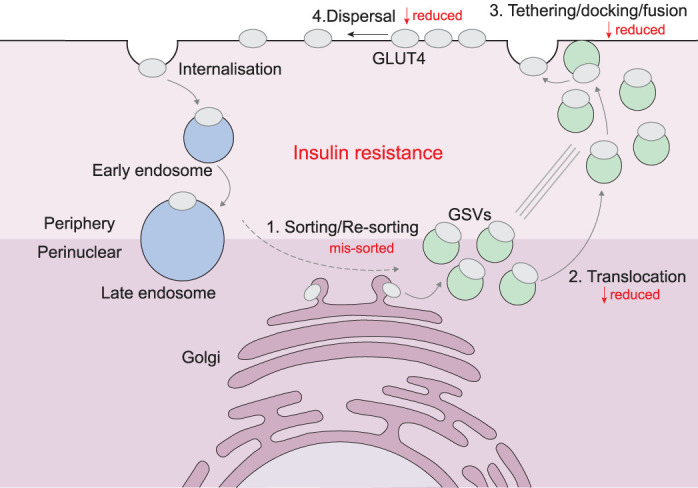
GLUT4 trafficking pathway. There are multiple steps in the GLUT4 trafficking pathway that could be impaired in insulin resistance: (**1**) Newly synthesised or internalised GLUT4 could be mis-sorted away from GSVs [4,5,48]; (**2**) Translocation of GSVs to the periphery of the cell in response to insulin could be reduced, such that there is less GLUT4 available for insertion into the membrane [57]; (**3**) Tethering, docking and fusion of GSVs may be reduced such that less GLUT4 is inserted into the membrane [58]; (**4**) Dispersal and density of GLUT4 in the membrane may be reduced [60]. Either one or a combination of these defects could result in reduced plasma membrane GLUT4 in response to insulin.

Insulin resistance is characterised by reduced cell-surface GLUT4 in response to insulin. The trafficking itinerary that moves GLUT4 to and from the cell surface is complex, involving a series of overlapping processes that (1) render GLUT4 insulin responsive by sorting it into GSVs, and (2) promote GLUT4 accumulation at the PM in adipocytes or sarcolemma and t-tubules in muscle in response to insulin signalling (Figure 3). Evidence suggests that this trafficking pathway itself is a crucial determinant of insulin resistance meriting independent characterisation. Firstly, the reduced insulin-stimulated cell-surface GLUT4 is generally due to an impairment in GLUT4 trafficking rather than a simple reduction in total GLUT4 levels. While reduced GLUT4 abundance has been observed in insulin-resistant human adipose tissue [39], the same was not true in skeletal muscle [40–43] despite impaired insulin-stimulated glucose transport. Additionally, studies in cultured cells [24,29], preclinical models [44], and humans [4] found that, even in insulin-resistant adipose tissue, there was substantial dysregulation of GLUT4 traffic. Secondly, impaired GLUT4 translocation may be independent, in part, from changes to the insulin signalling nodes that control GLUT4 trafficking. In particular, in insulin-resistant human muscle biopsies, hypoxia and AICAR-stimulated GLUT4 translocation *ex vivo* was impaired, even though these stimuli do not engage the classical insulin signalling pathway [45,46]. These findings suggest that studying GLUT4 trafficking itself will provide unique insight into the aetiology of insulin resistance.

### GLUT4 trafficking to an insulin-responsive compartment

Before insulin stimulation, GLUT4 is sorted away from other secretory cargo in the Golgi and packaged into small, insulin-responsive GSVs that contain a defined proteome (Figure 3) [47]. Mis-sorting or failure to generate GSVs may result in GLUT4 entering a non-insulin responsive compartment preventing insulin-stimulated GLUT4 translocation. Subfractionation studies in skeletal muscle or adipocyte biopsies from insulin-resistant and type 2 diabetic humans showed higher levels of GLUT4 in denser fractions compared with healthy controls [4,5,48]. GSVs generally fractionate into low density fractions due to their small size [49], as such these data may represent mis-sorting of GLUT4 away from GSVs. This could be responsible, in part, for the insulin resistance observed in these studies. The concept that GSV generation is a key determinant of insulin sensitivity is supported by data from exercised muscle. Increased GLUT4 translocation responses in human muscle post-exercise was accompanied by altered GLUT4 distribution in myofibres [50] and increased GLUT4 within GSVs as measured by colocalisation of GLUT4 with the GSV v-SNARE VAMP2 [51].

It is challenging to explain what could cause GLUT4 mis-sorting in insulin resistance as our understanding of GLUT4 sorting is far from complete. Nevertheless, there is evidence that known regulators of this process are altered in insulin resistance. For example, sortilin and STX16, proteins that facilitate sorting of GLUT4 to GSVs following its synthesis or internalisation from the PM, had altered abundance in insulin-resistant human skeletal muscle [52] and cultured myotubes [53]. Alternatively, changes in the Golgi lipid environment may contribute to impaired GLUT4 sorting. Ceramide accumulation in muscle and adipose tissue has been long proposed to drive insulin resistance [54], and ceramides can induce Golgi fragmentation and block ER-to-Golgi transport [55]. Furthermore, treatment of GLUT4-overexpressing L6 myoblasts with C2-ceramide impaired insulin-sensitive GLUT4 re-exocytosis after internalisation from the PM [56]. These data imply that cellular ceramides could promote insulin resistance through mis-sorting of internalised GLUT4.

### GLUT4 trafficking to and at the cell surface

Insulin redistributes GLUT4 from GSVs to the PM, which requires GSVs to be translocated to the cell surface, docked beneath, and subsequently fused with the PM (Figure 3). There is an increasing availability of techniques to specifically study these PM GLUT4 processes, which have revealed that these processes can be impaired in insulin resistance. For example, total internal reflection fluorescence microscopy (TIRFM) revealed that GSV accumulation under/at the PM was impaired in hyperinsulinemia-induced 3T3-L1 adipocyte insulin resistance, while GSV fusion with the PM appeared unaffected [57]. In contrast, TIRFM analysis of insulin-resistant primary human adipocytes uncovered reduced GSV tethering/docking at the cell surface, and reduced GSV fusion [58]. Finally, recent studies using dSTORM (direct stochastic optical reconstruction microscopy) found that dispersal of GLUT4 through the PM was reduced in hyperinsulinemia-induced 3T3-L1 adipocyte insulin resistance [59,60]. These findings are particularly exciting since GLUT4 dispersion is largely uncharacterised, and merits further investigation to determine its functional significance.

One possible explanation for altered GSV interactions with the PM in insulin resistance is changes in the abundance of proteins comprising the SNARE complex and/or regulators of SNARE complex formation. VAMP2, VAMP3, and STX4 were elevated in skeletal muscle tissues from zucker diabetic fatty rats [61] while VAMP5 and SNAP29 were increased in insulin-resistant cardiac tissue [62]. Since fusogenic SNARE complexes require specific subunit stoichiometry, altered SNARE protein abundance could lead to ‘dead-end’ complexes that are unable to facilitate fusion. Munc18c, which binds to STX4 to negatively regulate complex formation, was also reported to have higher mRNA and protein abundance in skeletal muscle from insulin-resistant mice and insulin-resistant C2C12 myocytes [63]. Together, these changes in regulators of GSV fusion at the PM may contribute to lower GLUT4 cell surface levels in insulin resistance.

### Possible role of the cytoskeleton in impaired GLUT4 translocation

Insulin acutely remodels both the actin [64,65] and microtubule cytoskeletons [66–68]. Furthermore, pharmacological and/or genetic approaches implicate both actin [65,67,69–72] and microtubules ([73–76] and reviewed in [77]) in insulin-stimulated GLUT4 traffic. Microtubule and actin remodelling is thought to promote GSV delivery to the periphery [76,78], and augment GSV-PM fusion [64,79,80], respectively.

Loss of cortical actin or disruption of actin remodelling has been reported in multiple models of insulin resistance, including cultured mouse adipocytes and myotubes, and mouse and human skeletal muscle [65,81–83]. Studies in cultured myocytes, mouse skeletal muscle, and human muscle biopsies suggest that the regulation of actin remodelling by β-catenin [84,85] and Rac1 [38,82] is impaired in insulin resistance. Furthermore, studies in mouse and human skeletal muscle suggest loss of cortical actin arises due to increased cholesterol at the PM [86,87]. However, we note that the *in vivo* contribution of actin remodelling to skeletal muscle GLUT4 translocation may not be as great as observed *in vitro*, since muscle-specific knockout of both β- and ɣ- actin isoforms in adult mice had limited impact on insulin-stimulated glucose transport [88,89].

In addition to changes in actin, insulin-resistant skeletal muscle displayed impaired microtubule polymerisation and microtubule-based GLUT4 trafficking *in vitro* (ceramide treatment) and *in vivo* (diet-induced obesity) [75]. Potential mechanisms include altered microtubule-GSV interactions (possibly via impaired GSV tethering to the motor KIF5B [90]), insulin-regulated microtubule dynamics (possibly through altered MARK kinase activity [34], as described above) or microtubule function (possibly via ɑ-tubulin post-translational modification such as K40 acetylation [91]).

## Insulin signalling to GLUT4 traffic

There are multiple sites at which insulin signalling targets GLUT4 traffic [92], providing several channels through which dysregulated signalling can propagate to impaired traffic. The most direct of these is the Akt-TBC1D4 axis, as several pieces of evidence obtained in 3T3-L1 adipocytes indicate that this axis is critical for insulin-stimulated GLUT4 translocation. First, Akt activation by drug-induced membrane localisation was sufficient to fully induce GLUT4 translocation [93]; second, Akt inhibitors completely abrogated insulin-stimulated GLUT4 translocation [94]; third, TBC1D4 is an Akt substrate rapidly phosphorylated in response to insulin [95]; and finally, overexpression of a phospho-dead mutant of TBC1D4 (mutation of the Akt phosphosites S318, S588, T642, and S751 to Ala) impeded insulin-stimulated GLUT4 traffic in a dominant-negative manner [96]. The site at which TBC1D4 acts in GLUT4 traffic is less clear, with TBC1D4 implicated in both GSV biogenesis and retention in the perinuclear region [97] and in GSV interactions with the PM ([98] and reviewed in [92]).

The key role that TBC1D4 plays in regulated GLUT4 traffic has rendered it of major interest in insulin resistance. Indeed, insulin-stimulated phosphorylation of multiple TBC1D4 phosphosites was impaired in insulin-resistant muscle from diabetic or TNFα-infused patients [99,100], and conversely, exercise-induced phosphorylation of TBC1D4 S711 was required for improved insulin action in mice [101,102]. However, a number of studies reported no impairment in insulin signalling to TBC1D4 in insulin-resistant cells, mice, and humans [16,23,24,28,29,57]. These studies quantified total Akt-mediated phosphorylation of TBC1D4 using an antibody recognising the phosphorylated Akt consensus motif [23], or an antibody recognising phosphorylated TBC1D4 T642 [16,24,28,29,57]. Individual mutagenesis of T642 and S588 demonstrated that the former plays a substantially greater role in GLUT4 translocation [96]. Hence, it will be necessary to dissect the individual and combinatorial contributions of all insulin-regulated TBC1D4 phosphosites to GLUT4 trafficking, to decipher the extent to which intact and impaired TBC1D4 phosphorylation modulates insulin resistance.

The recent phosphoproteomics studies discussed earlier in this review can facilitate more global examination of dysregulated signalling-to-trafficking axes. Mining these data reveals that many known regulators of GLUT4 traffic harbour insulin-regulated phosphosites that are dysregulated in insulin resistance (Table 1). For example, given the dysregulation of PM GLUT4 processes in insulin resistance described above, future studies could focus on phospho-dysregulated mediators of GLUT4 traffic at the PM such as EFR3A, PI4K, and Cavin proteins (Table 1). Additionally, phosphoproteomics and subsequent experiments using kinase inhibitors demonstrated that the failure of insulin to deactivate GSK3 in adipocyte insulin resistance was partially responsible for impaired GLUT4 translocation [34]. In a healthy state, GSK3 likely modulates multiple arms of GLUT4 traffic, as it has recently been shown to regulate GLUT4 translocation through the GSV-localised protein TRARG1 in cultured adipocytes [103], and GLUT4 endocytosis through dynamin-2 in cultured myoblasts [104]. Furthermore, as discussed above, the canonical GSK3 substrate β-catenin can regulate GLUT4 translocation through cortical actin remodelling [85,105], and this mechanism may be impaired in insulin resistance [84]. Finally, unbiased phosphoproteomics combined with kinase inhibition has revealed hundreds of novel GSK3 substrates [34], many of which have known or plausible roles in GLUT4 traffic including the regulator of GLUT4 endocytosis GAPVD1/GAPEX5 (GSK3 sites: S758 and T762 in mice) [106–108], the early/recycling endosome regulator RBSN/Rabenosyn-5 (S225) [109], and the endosome-to-Golgi trafficking regulator GOLGA4/golgin-245 (T39 and S40) [110]. These findings underscore the power of phosphoproteomics to systematically uncover novel intersections between insulin signalling and GLUT4 traffic. Functional studies are now needed to pinpoint whether and how these signalling pathways regulate GLUT4 traffic and contribute to insulin resistance.

**Table 1:** Insulin resistance alters insulin-regulated phosphorylation of GLUT4 trafficking mediators

GLUT4 trafficking process	Insulin-regulated phosphoproteins in Fazakerley et al. [34] (adipocytes)			Insulin-regulated phosphoproteins in Haider et al. [33] (myocytes)		
	Unaffected insulin regulation	Emergent	Defective	Unaffected insulin regulation	Emergent	Defective
Formation of the IR-IRS-PI3K complex	Irs1, Irs2	Insr, Irs1, Irs2	Insr, Irs1, Irs2, Pik3c2a	IRS1, IRS2		
Akt activation	Akt1, Akt2, Akt3, Mtor, Pdpk1, Prr5, Rictor	Akt2, Prr5	Deptor, Rictor	AKT1	MTOR	
Actin remodelling	Ehbp1, Ehd2, Sorbs1	Ehbp1, Micall2, Sorbs1	Ehbp1, Sorbs1			
Translocation of GSVs to PM and tethering of GSV at PM	Dennd4c, Exoc4, Myo5a, Rab3d, Ralgapa2, Sec16a, Tbc1d4	Camk2d, Camk2g, Dennd4c, Ralgapa2, Sec16a, Tbc1d4	Tbc1d4	TBC1D4		
GSV docking, fusion with PM and dispersal of GLUT4 in the PM		Efr3a, Stxbp5	Pi4ka, Stxbp5			
GLUT4 internalisation (endocytosis)	Cav1, Cavin2, Ehd2, Trip10	Cavin1, Cavin2, Cltc	Cav1			
GLUT4 return to GSV compartment and sorting away from recycling endosomes	Gga2, Ist1, Kif13b, Rab35, Snx2, Trarg1	Kif13a, Stx16, Trarg1, Vps26b, Vps35	Kif13b, Snx2, Trarg1, Vps26b			
Delivery of newly synthesised GLUT4 to GSVs and maintaining insulin responsiveness	Gga2, Sec16a	Axin1, Sec16a, Stx16				

Two phosphoproteomics studies of insulin resistance in adipocytes [34] and myocytes [33] were queried for insulin-regulated phosphosites on regulators of GLUT4 traffic collated from [92]. The indicated proteins harbour phosphosites that are unaffected in insulin resistance, emergent in insulin resistance (Fazakerley et al. [34]: ‘emergent' in ≥1 insulin resistance model; Haider et al. [33]: ‘Class 1B' and ‘Class 1C' phosphosites), or defective in insulin resistance (Fazakerley et al. [34]: ‘defective' in ≥1 insulin resistance model; Haider et al. [33]: ‘Class 1D' phosphosites).

## Looking to the future

The rise of phosphoproteomics and of techniques to study distinct aspects of GLUT4 traffic have provided strong leads in deciphering how the insulin-GLUT4 pathway is impaired in insulin resistance. This includes the identification of new kinases and regulated phosphosites that may play a role in insulin-regulated GLUT4 traffic, and clear evidence that GSV-PM interactions are altered in insulin resistance. Below we discuss some of the remaining barriers that limit our understanding of insulin signalling and GLUT4 traffic impairment in insulin resistance.

### Assessment of phosphosite function

One of the key limitations of the phosphoproteomics studies discussed above is that most of the several hundred dysregulated phosphosites detected lack functional characterisation. Hence, we face the daunting task of decoding which of these contribute to impaired glucose metabolism in insulin resistance, whether multiple sites act in concert, and how they impinge on GLUT4 traffic. Methods have been developed to systematically screen phosphosite function by mutagenesis in yeast [111], and the creation of similar methods for mammalian systems would greatly aid this task. On the other hand, bioinformatics models have been developed to predict the functional potential of individual phosphosites [112]. Development of models to predict specific phosphosite functions, such as regulating enzymatic activity or protein–protein interactions, would streamline efforts to pinpoint key changes in signalling that impair GLUT4 traffic in insulin resistance.

### Studying GLUT4 traffic

Only few studies have examined distinct GLUT4 trafficking processes specifically in insulin resistance, as it has been traditionally difficult to study these processes separately due to the heterogeneous distribution of intracellular GLUT4. Advances in TIRFM have substantially improved our ability to examine PM GLUT4 processes, leading to observations of their impairment in insulin resistance as discussed above. We anticipate that the development of new technologies will similarly facilitate the examination of intracellular GLUT4 trafficking stages such as sorting into GSVs — a step that may be impaired in insulin resistance (Figure 3). For instance, the Retention Using Selective Hooks (RUSH) system [113] may permit analysis of GLUT4 traffic through intracellular sites like the Golgi and GSVs. Finally, it will be necessary to perform integrative studies examining all aspects of GLUT4 traffic in appropriate model systems, to comprehensively chart its dysregulation in insulin resistance.

### Model systems

Indeed, a key consideration for studies of both trafficking and signalling in insulin resistance is the model system used. Muscle insulin resistance is of particular interest as muscle is the site of greatest postprandial glucose disposal [114]. While *in vitro* models allow more controlled and complex study designs compared with *in vivo* models, cultured muscle cell models typically have low endogenous GLUT4 expression [115,116], often lack a mature transverse tubule system [117], and do not recapitulate *in vivo* insulin responses to the same degree as the 3T3-L1 adipocyte. However, it is necessary to directly study muscle cells, since there are key differences in the signalling and trafficking machinery between adipocytes and myocytes, for example in the major Rab proteins responsible for insulin-regulated GLUT4 delivery to the cell surface (Rab8a and 13 in muscle [118]; Rab10 in adipocytes [119]) and in some signalling elements (e.g. Rac1 signalling in muscle [37]). The iMyos cells employed by Batista, Haider and colleagues provide an elegant means to profile signalling responses from individuals [32,33], however the increase in glucose uptake stimulated by insulin is far below that observed in skeletal muscle *in vivo* [120–122], suggesting this model may not recapitulate important aspects of muscle insulin action. Additionally, while 3T3-L1 adipocytes have been a workhorse cell line for studying GLUT4 biology, they are immortalised mouse cells, and the field would benefit from more translational adipocyte models, such as human-derived lines or tissues. Studying GLUT4 itself in more challenging samples such as patient-derived cells lines and tissues has been made more feasible through the recent development of antibodies that recognise extracellular epitopes on GLUT4 [123,124], meaning that PM abundance of endogenous GLUT4 can be assessed without the need for genetically engineered epitope tags.

## Conclusions

Impaired insulin-stimulated translocation of GLUT4 to the cell surface is the major defect in muscle and adipose tissue insulin resistance, leading to dysregulated glucose homeostasis and predisposing individuals to type 2 diabetes. Despite this, there are no existing therapies for type 2 diabetes, metabolic disease, or insulin resistance that specifically target GLUT4. Developing such therapies necessitates a detailed understanding of the cellular machinery that choreographs GLUT4 movement throughout the cell and, critically, how this machinery is altered in insulin resistance. Phosphoproteomics has revolutionised our understanding of the signalling changes occurring in insulin resistance, revealing complex rearrangements rather than simple defects in insulin signalling. Furthermore, evidence shows that distinct stages of GLUT4 trafficking are altered in insulin resistance, but further insight into the specific impairments of each stage is needed. Ultimately, we still have much to learn about how insulin signalling manipulates GLUT4 trafficking and how this becomes dysregulated in insulin resistance. This will likely require integrating methods to manipulate specific phosphosites, kinases, or phosphatases with assays examining specific GLUT4 trafficking processes. Understanding the interface between signalling and trafficking offers the best potential for developing effective therapies targeting insulin resistance.

## Perspectives

- Insulin resistance in muscle and adipose tissue is defined by impaired insulin-stimulated translocation of GLUT4 to the PM and an associated reduction in glucose uptake. Insulin resistance is an important driver of metabolic disease, yet the molecular changes in insulin signalling and/or GLUT4 traffic that confer insulin resistance remain largely unknown.
- Developments in phosphoproteomics and methods to interrogate GLUT4 traffic have revealed alterations in both insulin signalling and specific stages of the GLUT4 trafficking pathway in insulin resistance.
- Pairing phosphoproteomics with dissection of specific GLUT4 trafficking processes will prove useful in understanding the signalling-trafficking interface and mechanisms of insulin resistance.

## Open Access

Open access for this article was enabled by the participation of University of Cambridge in an all-inclusive *Read & Publish* agreement with Portland Press and the Biochemical Society under a transformative agreement with JISC.

## Abbreviations

GSVs: GLUT4-storage vesicles
PM: plasma membrane
TIRFM: total internal reflection fluorescence microscopy
